# Convenient non-invasive electrochemical techniques to monitor microbial processes: current state and perspectives

**DOI:** 10.1007/s00253-019-10091-y

**Published:** 2019-08-28

**Authors:** Charles E. Turick, Sirivatch Shimpalee, Pongsarun Satjaritanun, John Weidner, Scott Greenway

**Affiliations:** 1 Savannah River National Laboratory, Environmental Science and Biotechnology, Aiken, SC USA; 2 Department of Chemical Engineering and Computing, University of South Carolina, 541 Main Street, Columbia, SC USA; 3 Savannah River Consulting, 301 Gateway Drive, Aiken, SC USA

**Keywords:** Bioprocess, In situ monitoring, Cyclic voltammetry, Electrochemical impedance spectroscopy, Microbial fuel cell, Bioelectrosynthesis

## Abstract

Real-time electrochemical monitoring in bioprocesses is an improvement over existing systems because it is versatile and provides more information to the user than periodic measurements of cell density or metabolic activity. Real-time electrochemical monitoring provides the ability to monitor the physiological status of actively growing cells related to electron transfer activity and potential changes in the proton gradient of the cells. Voltammetric and amperometric techniques offer opportunities to monitor electron transfer reactions when electrogenic microbes are used in microbial fuel cells or bioelectrochemical synthesis. Impedance techniques provide the ability to monitor the physiological status of a wide range of microorganisms in conventional bioprocesses. Impedance techniques involve scanning a range of frequencies to define physiological activity in terms of equivalent electrical circuits, thereby enabling the use of computer modeling to evaluate specific growth parameters. Electrochemical monitoring of microbial activity has applications throughout the biotechnology industry for generating real-time data and offers the potential for automated process controls for specific bioprocesses.

## Introduction

The biotechnology industry includes microorganisms involved in environmental and waste cleanup, production of fuels and chemicals, as well as pharmaceuticals and food. Biotechnology is a significant part of the international economy with estimated revenues in the USA alone in 2012 of at least $350 billion or > 2.0% GDP (Carlson 2016). Industrial biotechnology relies on living microorganisms that convert a raw material to a product. Throughout this process, microorganisms and their chemical byproducts need to be sampled and analyzed for quality control purposes. Sampling and analysis however is time consuming, labor intensive, and costly, forcing a minimalistic approach to data gathering to keep costs down. This necessity brings with it a dilemma, data gaps that can lead to expensive misinterpretations of the status of a bioprocess. In situ monitoring using electrochemical techniques like chronoamperometry (CA), cyclic voltammetry (CV), and impedance techniques like electrochemical impedance spectrometry (EIS) are gaining use in chemical processing (Guth et al. 2009) and offer a solution to this dilemma in biotechnology as well by providing an abundance of real-time data at lower costs, because sample collection and off-line monitoring are not required.

**Fig. 1:**
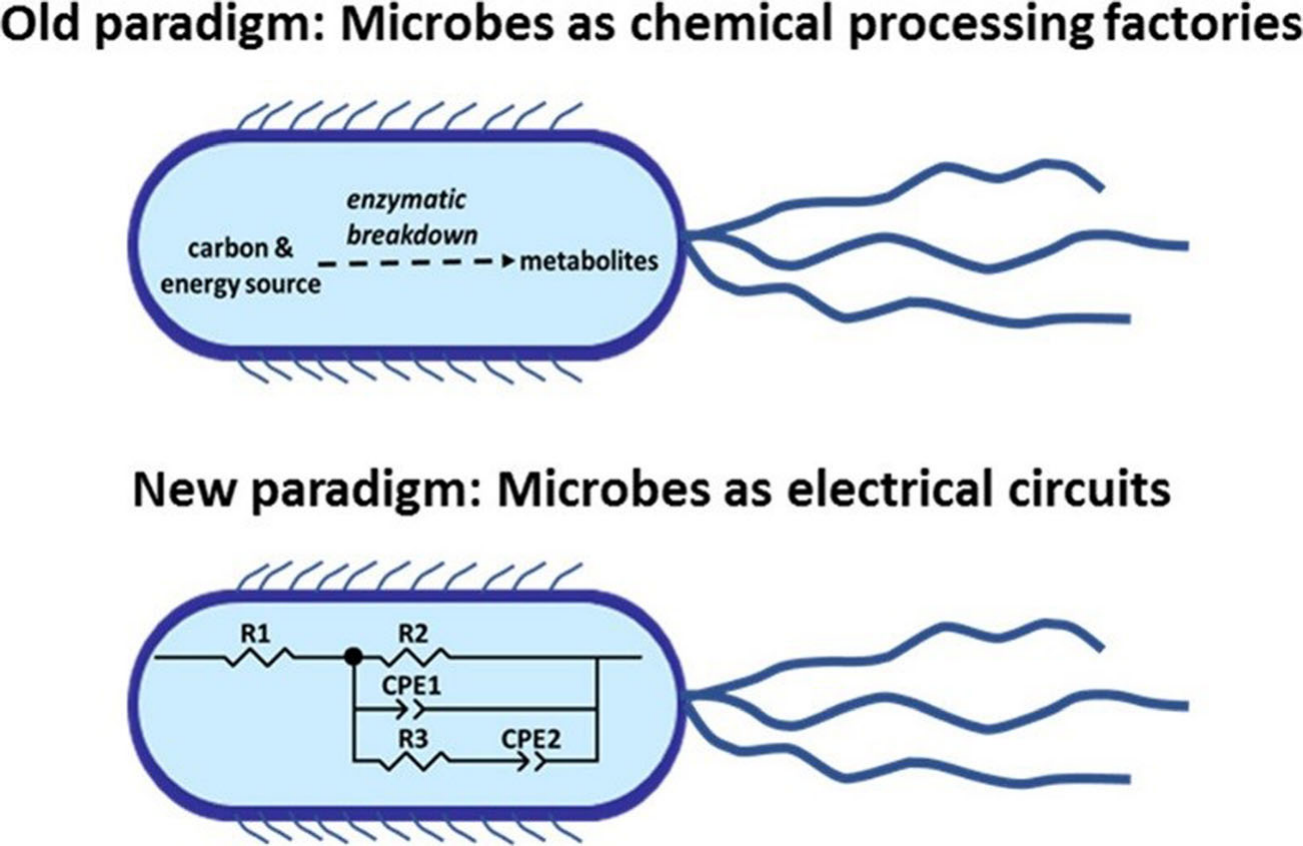
Changing paradigm from microbes as chemical catalysts to microbes as complex electrochemical entities. Since microbes are responsible for electron flow during catalytic activity, monitoring electron flow offers real-time data related to bioprocess status. With electrochemical techniques, microbial activity can be monitored continuously and inexpensively in real time. Potential disruptions can be detected early and corrected before process failure occurs.

The acquisition of abundant, real-time data at low cost for biotechnology requires a century-old paradigm to shift (Fig. 1). Typically, microbes have been viewed as chemical catalysts and monitored using physical and chemical methods. If there is a disturbance in a bioprocess (i.e., changes to aeration, pH, temperature, and contamination), it can cause a decrease or a halt in microbial activity and affect chemical catalysis. This perturbation will show up eventually in the chemistry data as the evidence gradually builds up to show a decrease in product (and/or increase of an inhibitor). While this approach is rational, the chemical changes being catalyzed by microbes are driven by electron flow for cellular health, replicatio, n and chemical production. If one views microbes as electrical circuits as well as chemical catalysts, a whole new arsenal of techniques can be used to follow their activity, in real time, throughout growth. In this respect, actively metabolizing microbes can be viewed as sensors since the structural and functional changes that occur to the cell during growth can be detected electrochemically. This approach can be useful in physiological studies as well as monitoring bioprocesses in real time, with the potential to link monitoring to automated bioprocess control. The challenge is to link the electrochemistry to the biology.

### Microbes as electrochemical entities

The convergence of electrochemistry and microbiology is significant as it has launched the tremendous field of biosensing. Biosensors are defined as chemical sensors where the recognition system uses a biochemical mechanism (Cammann 1977; Thévenot et al. 2001) and the resulting signal is transferred to the electrical domain (Thévenot et al. 2001). Biosensors utilize biocatalysts from three categories: enzymes, plant or animal tissues, and whole cells. The overall topic of biosensor technology has received several excellent reviews (Ali et al. 2017; Thévenot et al. 2001; Vigneshvar et al. 2016). This mini-review is directed primarily to professionals involved in bioprocess technologies and summarizes the current state of electrochemical techniques for monitoring the physiological status of whole microorganisms with representative examples from the literature. Topics covered include amperometric and voltammetric approaches related primarily to microbes capable of electron transfer to and/or from electrodes (electrogenic microbes). Impedance techniques are also covered and include capabilities for monitoring cell density, viability, and physiological status of microbial cultures.

In the early twentieth century, yeast and bacteria were first shown to interact with electrodes by generating a current during growth (Potter 1911; Cohen 1931). Later in the century, the discovery of bacteria capable of growth by extracellular electron transfer (EET) to mineral oxides (electrogenic microorganisms) (Lovley et al. 1998; Myers and Nealson 1988) led to a renewed interest in microorganisms as electroactive entities. With this process, organic carbon or hydrogen serves as the electron source for metabolic energy with EET to a solid terminal electron acceptor (mineral oxide or electrode) occurring through direct contact of the cell to the electrode or with the use of redox mediators such as riboflavin, humics, anthraquinone 2,6 disulfonate, and melanin (Lovley et al. 1996; Marsili et al. 2008; Turick et al. 2002). Applications were initially directed to bioremediation of heavy metal contamination and eventually evolved into microbial electron transfer to electrodes. A considerable amount of the latter endeavors focused on electricity production from microbial fuel cells, mostly for use as bioprocesses for conversion of waste to energy will be discussed below.

Electron transfer from electrodes to microorganisms is also an emerging field that includes applications for contaminant conversion as well as chemical production (bioelectrosynthesis) and will also be discussed below. Electrochemical techniques are also being adapted to the study of microbial physiology. Much of this work has incorporated electrochemical techniques and provided important information about the capabilities of various microbial types and EET mechanisms involving a wide variety of cell structures and physiologies (Kracke et al. 2015; Lovley 2012; Nealson and Rowe 2016; Obi and Asogwa 2015). This incorporation of techniques in electrochemistry for the study of microbial physiology provides novel opportunities to study electron transfer phenomena in real time as well as applying these principles to bioprocess monitoring.

### Chronoamperometry

**Fig. 2:**
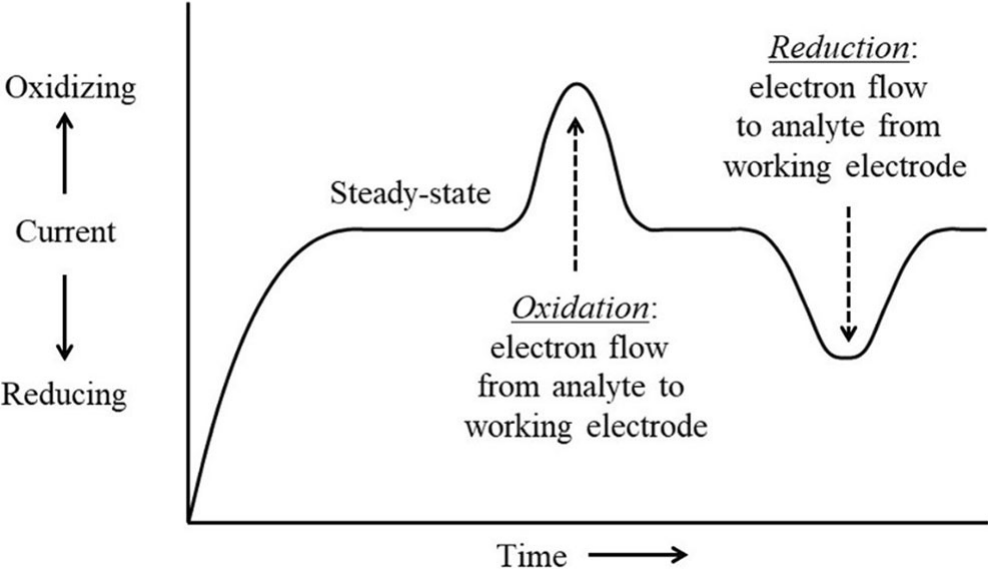
Plot of chronoamperometry data. Application of chronoamperometry in microbial cultures provides quantitative information regarding electron transfer to and from electrodes

Chronoamperometry (CA) is an electrochemical technique for monitoring changes in current with time during the application of a constant potential or after a step-change in potential. Initially, during a CA study, the current will decay after the change in voltage to a steady-state current. CA is a commonly used electroanalytical technique to determine diffusion coefficients and for investigating kinetics and mechanisms of electron transfer. For electrochemists, CA is often used to determine transient values shortly after a step-change in potentials, and to characterize the steady-state response after reactive currents have decayed and after mass transfer limitations have manifested themselves. This can provide information about the long-term steady-state reaction rate expected at a specific condition. Regarding electroactive microorganisms, CA allows the working electrode (WE) to serve as a terminal electron acceptor when poised at a desired oxidation potential, while electron flow from the microbes to the electrode is detected over time as an increase in current (Fig. 2). In this case, the WE serves as the anode. This provides a real-time method to quantify EET and is often a function of the availability of a carbon and energy source.

Along the same line, if the electrical potential of the WE is poised at reducing conditions, electron flow to microbes from the WE can also be studied (Fig. 2). This has applications for driving metabolic activity (bioelectrosynthesis) of microbes in contact with the working electrode, through external reducing power from electron flow to the cells and will be discussed below.

While much of the research regarding microbial mechanisms of EET has focused on two model electroactive genera of Gram-negative bacteria (*Shewanella* and *Geobacter*) (Kracke et al. 2015; Lovley 2012; Nealson and Rowe 2016), over time, a broad diversity of electroactive microbes are being realized. As an example, chronoamperometry was used to quantify microbial metabolic activity relative to organic carbon availability to diverse microbial populations in sediments (Friedman et al. 2012; Wardman et al. 2014) as well as a Gram-positive thermophile (Mathis et al. 2008). In addition, chronoamperometry was used to monitor organic carbon utilization, temperature effects, and the requirement for redox mediators by a Gram-positive spore former (Milliken and May 2007). Current production was demonstrated from a wide variety of pure cultures that included the lactic acid bacterium *Lactobacillus sakei*, the yeast *Kluyveromyces marxianus*, and the basidiomycete *Pleurotus sapidus* (Pescheck et al. 2005). Using immobilized cells, current production mirrored cell density and coincided with carbon and energy source (glucose) utilization. Increased current was also detected with the human cell lines JURKAT and SBC-7, with higher current detected with N-methylphenazonium methyl sulfate as a redox mediator.

Application of chronoamperometry has demonstrated potential for monitoring subsurface microbial activity during bioremediation. A strong correlation to subsurface microbial electrogenic activity was demonstrated where acetate utilization corresponded quantitatively to current production (Wardman et al. 2014). Current production was higher in the presence of the naturally occurring terminal electron acceptors sulfate and Fe (III) compared with methanogenic conditions due to the reduced byproducts Fe (II) and sulfide. Field studies in uranium-contaminated groundwater showed increased current production from indigenous microbes associated with acetate amendments and uranium reduction (Williams et al. 2010). This study used graphite cylinders as electrodes in boreholes to depths of 6 m with current calculated through the voltage drop across a 560 Ω resistor at surface electrodes.

### Hydrodynamic chronoamperometry

Electrochemical monitoring of soluble redox mediators linked to microbial metabolic activity using conventional chronoamperometry can be limited by diffusional processes, mediator concentration gradients, and mediator reduction in the diffusion layer of the electrode. Hydrodynamic chronoamperometry incorporates a rotating disc electrode to alleviate these issues, resulting in accurate metabolic rates at short temporal resolution which allows for continuous monitoring of fast cellular events (Prévoteau et al. 2015). Applications that could benefit from this technique are rapid assessments of online fermentation processes and for biological oxygen demand (BOD) or toxicity.

**Fig. 3:**
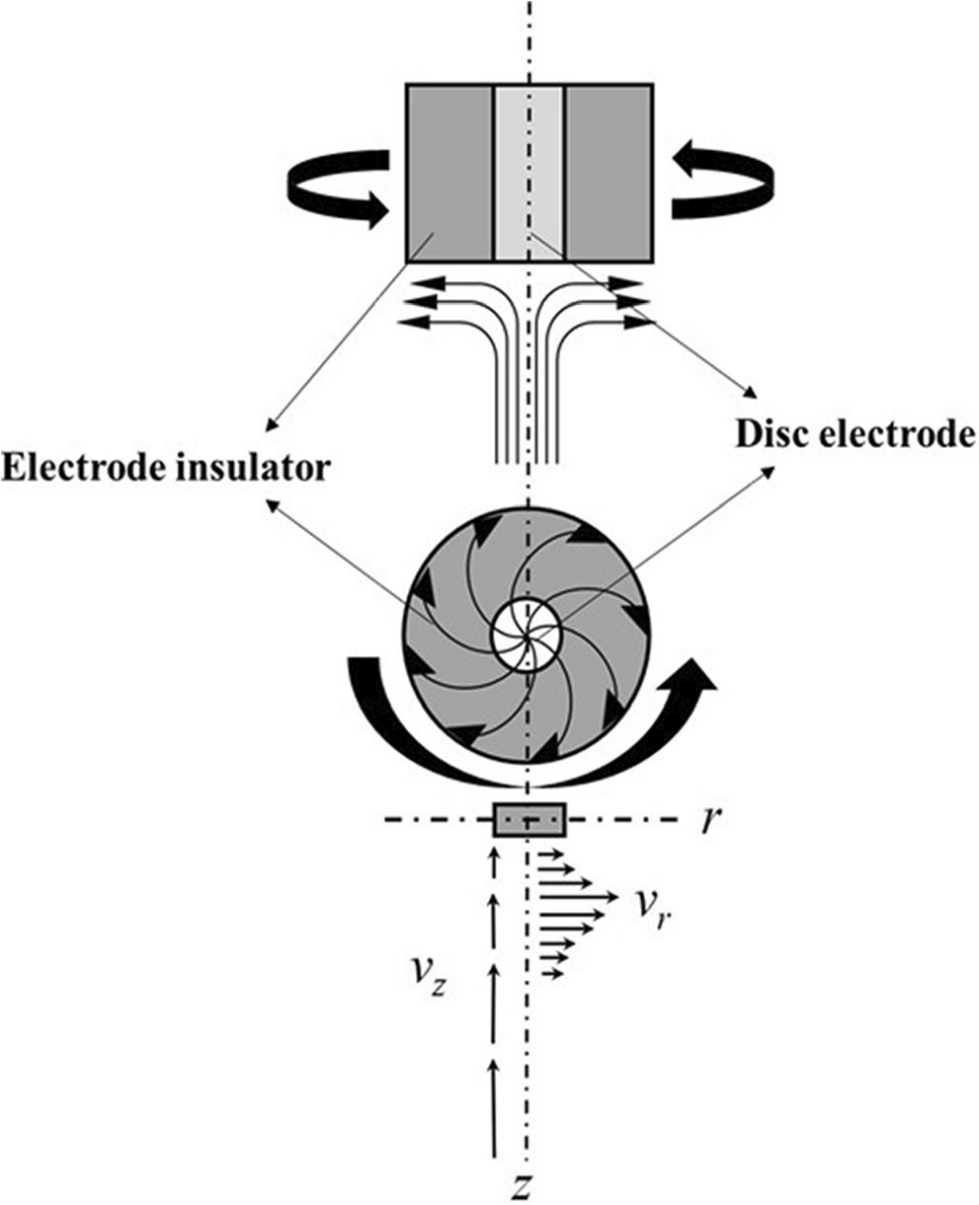
Streamlines for flow and vector of fluid velocity near a rotating disk electrode (adapted from Denuault et al. 2007, with permission)

The rotating disc electrode (RDE) is the classical hydrodynamic electroanalytical technique used to limit the diffusion layer thickness. The RDE uses the concept of forced convection that has several advantages which include (1) the rapid creation of a high rate of steady state mass transport and (2) simply controlled convection over a wide range of mass transfer coefficients. The RDE consists of a disc (e.g., of Pt, Ni, Cu, Au, Fe, Si, CdS, GaAs, glassy carbon, and graphite) set into an insulating polytetrafluoroethylene mount that surrounds the electrode. The electrode is rotated about its vertical axis as shown in Fig. 3, typically between 400 and 10,000 rpm. The theory for the hydrodynamics at the RDE assumes that the electrode is uniformly accessible and affords a precise and reproducible control of the convection and diffusion of reactant to the electrode. Therefore, RDE can be used to study the kinetic of interfacial process.

### Cyclic voltammetry

Cyclic voltammetry (CV) can be used to measure reduction/oxidation (a.k.a. redox) conditions in the bioreactor system as well as the electrochemical activities of bacteria, especially in relation to electron transfer phenomena in MFCs (Eggleston et al. 2008; Fricke et al. 2008; Rabaey et al. 2004). The basics of CV explain a typical electrochemical measurement circuit made up of an electrochemical cell, an adjustable voltage source, an ammeter, and a voltmeter. Typically, the three electrode systems of the electrochemical cell are the WE, reference electrode, and the counter (or auxiliary) electrode. Some systems have four electrodes where an additional electrode is the working sensing. The voltage source for the potential scan is applied between the WE and the counter electrode. The potential between the reference electrode and the WE is measured with the voltmeter, and the overall voltage is adjusted to maintain the desired potential at the WE with respect to the reference electrode. The resulting current flowing to or from the WE is measured with the ammeter. This process is usually repeated for a range of electrode potential.

Here is the standard procedure that can be used to perform a measurement for each point in the scan: (1) Select a potential (*E*) for reference electrode with respect to WE. (2) Adjust the voltage across the entire cell (counter electrode to WE) to get desired *E*. (3) Measure current. (4) Select (step) a new *E* and repeat procedure until the scan is finished. The procedure can be a single sweep between two potentials. If this is the case, this is called linear sweep voltammetry. The procedure may also be configured that when a certain potential is reached, the sweep is inverted. This is called cyclic voltammetry. This cycle may be repeated multiple times during an experiment. (5) Plot the results and derive parameters of interest from the data.

**Fig. 4:**
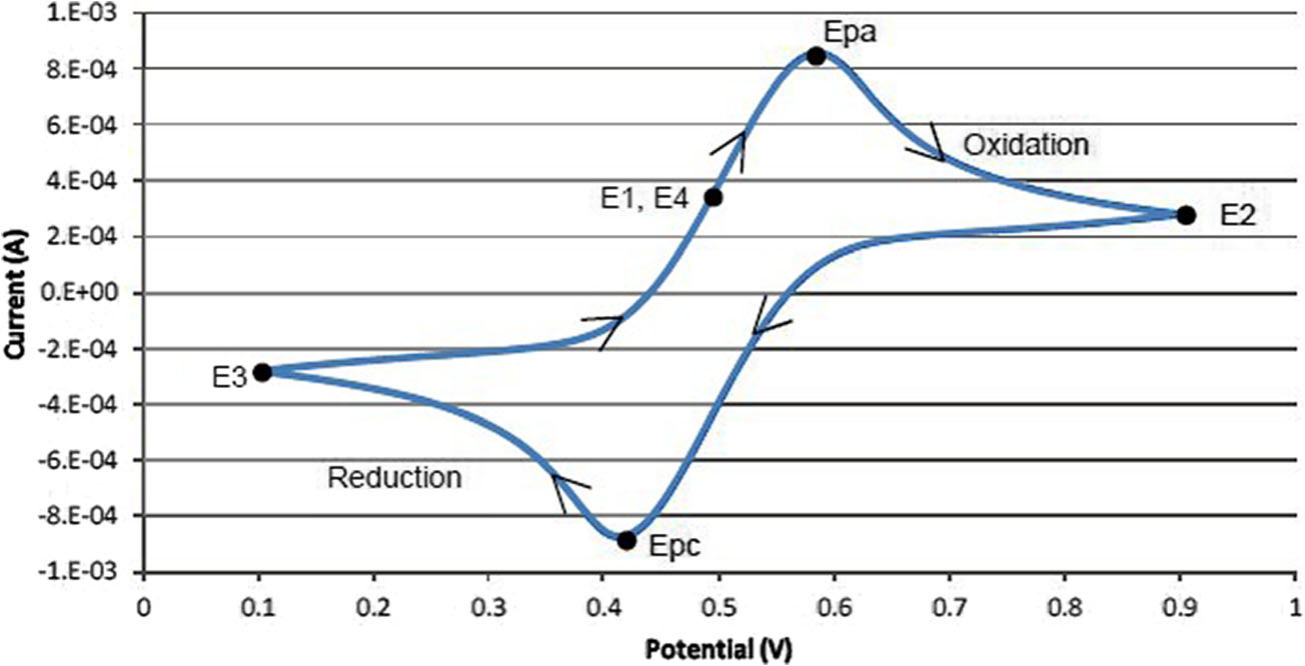
Example voltammogram (Tektronix 2016, with permission) depicting four voltage vertices: *E*1 (initial potential), *E*2 (second, switching potential), *E*3 (third, switching potential), and *E*4 (final potential). The voltage peaks in the waveform are the anodic (Epa) and the cathodic (Epc) peak potentials

Once the experiment is complete, the measured current is plotted as a function of the potential also known as a voltammogram. The example voltammogram is provided in Fig. 4. In this example, the scan begins at *E*1 and the potential becomes increasing more positive causing the anodic current to rise rapidly and peak at the anodic peak potential (Epa). At *E*2, the scan direction switches to negative for the reverse scan. As the current becomes more negative, cathodic current will flow as the electrode process is reduced. A cathodic peak potential occurs at Epc. At the third potential, *E*3, the direction is reversed again, and the voltage is swept until it reaches *E*4. From the potential sweep, important information about the experiment can be derived and analyzed. Harnisch and Freguia (2012) provide an excellent and more detailed description of CV applied to electroactive microbes.

As mentioned above, the CV data can provide information about redox potentials and electrochemical reaction rates. As early as 1984, Matsunaga and Namba used CV to detect and enumerate *S. cerevisiae.* They found that coenzyme A (CoA) concentrations in the cell wall changed as cell counts increased. These changes altered the electrochemistry of the medium, creating an oxidation peak in the voltammogram, and allowed the authors to indirectly calculate cell density. Additionally, the peak current changed and appeared to be closely related to cell viability and metabolism.

CV techniques offer considerable utility by providing the capability to quantify the rates of electron transfer (Turick et al. 2009) and differentiate between electrochemical interactions at microbial surface and the bulk phase (growth medium) (Laviron 1983; Turick et al. 2009) by correlating linearity of peak current to various scan rates and by employing rapid scan rates to repel biofilms for extended use in bioreactors (Martin et al. 2018).

CV techniques were also used to monitor actively growing cultures of *Clostridium phytofermentans*. These bacteria were prone to accept electrons from a reduced electrode during CV scans of 25 mV/s, but only when the carbon and energy source, cellobiose, was in decline (Martin et al. 2018). This approach was used to monitor the physiological status of the batch culture in terms of voltammetric charge density and was explained due to a likely imbalance in the internal NAD/NADH ratio, thereby making the cells more oxidized and hence prone to accepting external electrons.

Another useful parameter that CV can provide is to calculate is the “true” area of the electrode. In many cases, the geometric area of an electrode is not the same as the electrochemically active area, especially as a result of biofilm formation. Geometric area calculations assume a smooth surface, but any surface roughness increases the available area of the electrode. Electroactive surface area has been determined several ways (Trastatti and Petrii 1992). H_2_ or O_2_ adsorption onto metal electrodes is a common approach among electrochemists; however, these gases could react chemically with biofilms, and consequently other approaches are more desirable like the capacitance ratio or voltammetric techniques.

### Microbial fuel cells

Research directed at EET has led to the microbial fuel cell (MFC) for applications in bioprocessing of organics, such as waste to electricity using MFCs (Do et al. 2018; Kumar et al. 2018; Schaetzle et al. 2008). MFCs generate electricity by exploiting growth of electrogenic microbes in an anode chamber. There, the electron donner is oxidized and resulting electrons are transferred to the anode, which serves as the terminal electron acceptor. Electron flow passes through a circuit that includes an external resistance load on the way to the cathode where contact to a terminal electron acceptor is made (Drapcho et al. 2008). The variety of electrogenic microbes is contributing to the robustness of these applications (Do et al. 2018; Kumar et al. 2018; Schaetzle et al. 2008) with electron flow determined by specific metabolic activity (Biffinger et al. 2009; Holtman et al. 2006).

MFCs offer considerable potential as bioprocesses since they convert organics (often wastes) to electrical power with applications for wastewater treatment, remote power sources, biosensors, and water desalination, but are limited due to low power generation, expensive electrode materials, environmental conditions, substrate loading, scale up, and fuel cell configuration challenges (Krieg et al. 2019; Logan et al. 2015; Logan et al. 2018; Santoro et al. 2017; Slate et al. 2019; Wang et al. 2015;). These challenges are being addressed through bioprocess design and startup, as well as nanomaterials and genetically modified organisms for enhanced electron transfer, for more economical applications as energy-harvesting bioprocesses (Do et al. 2018; Kumar et al. 2018; Logan et al. 2015; Logan et al. 2018; Jiang et al. 2016; Santoro et al. 2017; Slate et al. 2019; Wang et al. 2015;). Successful field trials offer promise for this area of study, for example, maximum power to date of 70.8 mW/m^2^ and a COD reduction of 78–87% was demonstrated with an on-site MFC converting waste to energy (Dong et al. 2015; Ieropoulos et al. 2016).

An additional advantage of this technology is that real-time bioprocess monitoring occurs as a default since current production is the product and a measure of microbial activity. Hence, MFCs have been designed for online detection of biological oxygen demand (BOD) (Kharkwal et al. 2017; Zhou et al. 2017) as well as metal and contaminant detection (Pham 2018), including commercial applications (Kim et al. 2007). The potential for a self-powered bioprocess offers advantages for remote monitoring (Sun et al. 2015; Zhou 2015).

Another interesting application of MFC technology is microbial energy production linked to desalination (Kim and Logan 2013). This process works when a microbially generated potential gradient drives ion transport through ion exchange membranes (ibid.) Technical issues of membrane fouling with this technology have been addressed with the use of a sacrificial protective layer applied to the membrane (Son et al. 2018).

Microorganisms also demonstrate the ability to accept electrons from electrodes poised at reducing potentials, with metabolic activity being driven electrochemically. As an example, nitrate reduction was demonstrated using nitrate-reducing bacteria colonized onto carbon paper that served as a cathode (Kondaveeti and Min 2013). When a reducing potential was applied to the bacterial cathode, the degree of nitrate reduction was greater than sterile carbon paper and comparable with platinum. Intermediate products of nitrate reduction varied with reducing potential where 0.7 V yielded nitrite where ammonia predominated at 0.5 V and the biocathode showed a higher ratio of ammonia to nitrite compared with abiotic controls. Hence, the application of MFCs for contaminant removal, including metals, is another possible use for this technology, but first technical challenges including thermodynamic limits, biotoxicity, pH imbalance, and membrane biofouling need to be addressed (Ezziat et al. 2019).

MFC technology is also driving the field of bioelectrosynthesis which is showing considerable prospects as a bioprocess for the production fuels and chemicals, including electricity-driven reduction of CO_2_ (Alkotaini et al. 2018; Jiang and Zeng 2018; Kumar et al. 2018; Li et al. 2018; Rabaey and Rozenthal 2010). Advances in this field include increasing electron transfer efficiency and incorporating genetically modified microbes and bioprocess designs which offer potential in producing more valuable, higher carbon chain chemicals (Jiang and Zeng 2018).

### Impedance techniques

Electrochemical evaluation of microorganisms is not limited to those demonstrating EET. Living cells can be thought of as electrochemical entities that behave like small capacitors and can become polarized when exposed to a small electric field (Konakovsky et al. 2015; Slouka et al. 2016). In this case, cells can be described in terms of capacitance, electrical permittivity, and conductivity.

In a simple system, if the voltage (*E*) and current (*I*) are known, the resistance (*R*) can be calculated (*R* ≡ *E*/*I*). In a more complex system that includes an AC signal of known frequency and amplitude, *Z* ≡ *E*/*I*. *Z* is impedance and is a very complex version of resistance.

The change in properties of an AC signal at a specific frequency passing from one electrode to another and through environmental media (including microbes) furnishes impedance data. With EIS, a known potential is applied to a WE and the resulting current is measured at the counter electrode. The use of electrochemical impedance techniques to analyze microbial cells is possible because the signal reacts with components of living cells causing frequency-dependent polarization (Carvell and Dowd 2006; Slouka et al. 2016; Yardley et al. 2000). Signal responses can be related to the polar nature of lipid membranes, proton gradients on the outer surface of viable cells, and membrane-associated electron transfer reactions linked to metabolic activity. The cell surface is negatively charged due to the membrane glycoproteins that attract positive ions to the cell membrane forming a double layer. Additionally, the lipid bilayer of the cell and the ions contained within the cytoplasm allow the cell to interact electrochemically (Markx and Davey 1999). When an AC voltage is applied to cells, electrical conduction is faster through water than lipid bilayers, making the current lag behind the voltage and causing a phase shift. The phase shift, which is also frequency dependent, is measured geometrically as the angular transformation of the capacitance to resistance ratio, or phase angle (Cichoż-Lach and Michalak 2017). These parameters change throughout the cellular growth cycle and reflect factors related to biomass density, cell viability, membrane integrity, and the overall metabolic state of microbes (Busse et al. 2017; Guan et al. 2004; Kim et al. 2009). Cell size also plays a significant factor with greater sensitivity as a function of cell size (Markx and Davey 1999). Dead cells or non-biomass solids that do not pose an intact plasma membrane do not polarize and therefore do not have a significant capacitance relative to a cell suspension (Carvell and Dowd 2006).

Impedance techniques are also relatively insensitive to non-cellular matter (Patel and Markx 2008) and hence, are well suited to monitor living cells. Biological materials behave differently at different frequencies where their conductivity increases in a step-wise manner (known as dispersions) with increasing frequency while permittivity (a property of capacitance) decreases with increasing frequency. The steps are referred to as *α*, *β*, and *γ* dispersions, where *α*-dispersions are generally associated with the diffusion processes of ionic species due to the tangential flow of ions across cell surfaces. *β*-dispersions relate to interfacial polarization across the cellular plasma membranes and their interactions with the extra- and intra-cellular electrolytes, while γ-dispersions are at the higher range of frequencies in biological materials are due to the dipolar rotation small molecules, particularly water (Heileman et al. 2013; Markx and Davey 1999). The tremendous advantage of this label-free monitoring technique is that the inherent complexity of data across several logs of frequency offers the capability of delivering specific information regarding numerous multifaceted parameters, like changes in fluid composition (conductivity, reaction and diffusion rates), microbial density, metabolic activity, and growth status during each scan. These techniques offer substantial possibilities for monitoring bioprocesses online, in real time, to provide rapid evaluations of the stability of microbial populations in industrial bioprocesses with potential to revolutionize the field (Konakovsky et al. 2015).

The overall impedance is ǀZǀ with the impedance signal at the WE referred to as real impedance (*Z*′) and the signal at the counter electrode is the imaginary impedance (*Z*″). Cells in between the two electrodes can cause changes in the phase as well as the amplitude of the signal related to their physiological state.

The absolute magnitude of impedance can be expressed as:

$$ \mathrm{I}Z\mathrm{I}=\sqrt{Z^{\hbox{'}2}}+Z{{\prime\prime}}^2 $$

The phase angle (ϕ) can be defined by:

$$ \tan\ \upphi =Z^{{\prime\prime} }/Z^{\prime } $$

where,

IZI: absolute magnitude of impedance
*Z*′: real (in-phase) impedance
*Z*″: imaginary (out of phase) impedance
*ω*: angular frequency.

As the signal encounters cells, the phase and amplitude change relative to the physiological status of the cells. Impedance data can also be applied to design an equivalent circuit to model data from growing microbial cultures and relate the parameters to physiological status and even changes in the bulk chemistry of the growth medium. This aspect is also useful for monitoring changes in the bulk medium related to cellular byproducts. The use of a 100 kHz to 0.01 Hz range of frequencies provides data most useful for interests in biotechnology because the activities mentioned above fall into this range. This frequency range is sensitive to the double-layer capacitance of living cells and not affected by stirring or aeration of the culture (Slouka et al. 2017).

Capacitance (*C*) is the ratio of the change in an electric charge (*Q*) in a system to the corresponding change in its electric potential and expressed as:

$$ C=Q/\varDelta E $$

Capacitance of capacitor is the ability of a system to store an electric charge. It is expressed as the ratio of the electric charge on each conductor to the potential difference (i.e., voltage) between them. In an electrochemical system, the capacitance may occur because of the charge rearrangement at interfaces or of dielectric phenomena. The charge rearrangement takes place at an electrolytic double layer, so called, double-layer capacitance. An electrical double layer exists on the interface between an electrode and its surrounding electrolyte. This double layer is formed as ions from the solution adsorb onto the electrode surface. The charged electrode is separated from the charged ions by an insulating space, often on the order of angstroms. Charges separated by an insulator form a capacitor, so a bare metal immersed in an electrolyte will behave like a capacitor. It can be estimated that there will be 20 to 60 μF of capacitance for every 1 cm^2^ of electrode area though the value of the double-layer capacitance depends on many variables. Electrode potential, temperature, ionic concentrations, types of ions, oxide layers, electrode roughness, impurity adsorption, etc. are all factors.

Permittivity is a subset of capacitance and can be expressed in relation to capacitance as real (ε′) and imaginary (ε″) permittivity and defined as follows:

$$ \upvarepsilon^{\prime }=\frac{-Z"}{\left({Z^{\prime}}^2+Z{"}^2\right)\omega {C}_0} $$

$$ {\upvarepsilon}^{"}=\frac{-Z^{\prime }}{\left({Z^{\prime}}^2+Z{"}^2\right)\omega {C}_0} $$

where *ω* = angular frequency and *C*_0_ = capacitance of an empty cell.

Impedance can be viewed as a very complex type of resistance. In a simple DC system, resistance has an inverse relationship to conductivity. With impedance being similar to resistance, admittance (*Y*) is similar to conductivity and measured in Seamans. Imaginary admittance (*Y*″) because it is well correlated to cellular activity and is defined as:

$$ {Y}^{{\prime\prime} }=\frac{-Z^{{\prime\prime} }}{Z^{\prime 2}+Z{{\prime\prime}}^2} $$

*Y*″ can also be defined as Seamans/m, which is then called *σ*″ and defined as:

$$ \upsigma "=Y"\frac{d}{a} $$

with *d*= distance between electrodes and *a*= effective area of the electrode.

### Application of impedance techniques for biotechnology

Several examples of impedance techniques for monitoring microbial growth are presented here. EIS has been used to evaluate and refine the operation of MFCs. EIS, along with voltammetry techniques, was used to define the behavior of redox-active species produced by biofilms of *Geobacter sulfurreducens* on electrodes and revealed complexity of microbial outer membrane cytochromes (Marsili et al. 2008). EIS was also used as a non-intrusive technique to detect and evaluate microbially produced extracellular redox mediators (Ramasamy et al. 2009) as well as understanding the dominant kinetic resistance the anode in relation to overall power output (Ramasamy et al. 2008).

Impedance techniques also demonstrated utility in monitoring growth of lactic acid bacteria grown at 20, 40, 80, and 100 g/l glucose using both permittivity and conductivity measured at 5.7 MHz with a good correlation to biomass as measured by dry weight (Arnoux et al. 2005). Growth of *Escherichia coli*, *Pseudomonas putida*, *Bacillus subtilis*, and *Saccharomyces cerevisiae* was monitored with a wireless remote sensor that recoded permittivity data at 5.66–5.98 MHz and demonstrated increases of real and imaginary permittivity as a function of growth (Ong et al. 2001). *E. coli* also demonstrated a growth response as detected with real and imaginary permittivity relative to incubation temperatures of 25, 30, and 37 °C as well as a significant decline in permittivity values during incubation in a toxic CO atmosphere. The decline in permittivity at this frequency range was attributed to declines in polarization of the cells due to leaky membranes as a result of cell lysis.

Changes in non-ideal capacitance (as determined with a constant phase element) of yeast cultures in an industrial brewing process measured below 1 kHz demonstrated a strong correlation to cellular growth with glucose. However, utilization of maltose and ethanol demonstrated spikes in capacitance attributed to a maltose uptake mechanism that is different from that of glucose uptake, as well as a change in metabolism (Slouka et al. 2017). In this study, the idealized capacitance correlated well with cell density during growth on all carbon sources tested, demonstrating the utility of impedance techniques to monitor cell density as well as metabolic status of cells.

Industrial bioconversion of biomass to fuels and high-value platform chemicals often necessitates enzymatic pretreatment of the recalcitrant biomass to convert lignocellulosic material to simple sugars to accelerate bioconversion kinetics. Simultaneous saccharification and fermentation of lignocellulosic biomass was monitored at 580 kHz in real time and provided important kinetic data (as conductivity) regarding enzyme activity and associated appearance of organic acids. Microbial carbon source utilization was also detected demonstrating the potential of this technique for monitoring simultaneous saccharification and fermentation (Bryant et al. 2011).

Fed-batch cultures of *Bacillus thuringiensis* (Sarrafzadeh et al. 2005) were followed via permittivity (2 MHz) and correlated well with growth phases, including sporulation. The decline in permittivity after growth ceased was associated with the increase of spore formation. This demonstrated additional utility of real-time impedance techniques in that they can also be used to monitor physiological changes to the cells during the growth cycle.

In many industrial biotechnology applications that incorporate yeasts, flocculation behavior of the yeast cells is an important property and a function of cell surface charge. Kregiel et al. (2012) associated cell surface charge to flocculation capacity of several yeast strains by monitoring permittivity (1 kHz) of several industrial yeast strains with established flocculation behavior. Permittivity correlated well with flocculation ability and conventional methods to determine flocculation and demonstrated a strong negative correlation to capacitance. This approach is viewed as a potential rapid assay for yeast flocculation abilities.

Impedance analyses of microbial cultures can also incorporate an entire spectrum of frequencies to obtain information about various parameters simultaneously. Impedance data can also be analyzed across the entire spectrum of frequencies used at any given time. That involves the complex dielectric function (Cole and Cole 1942) presented as Bode plots where *Z*″ is plotted over *Z*′ or Cole-Cole plots where ε″ is plotted over ε′.

The resulting data from these analyses could then be interpreted using circuit models to determine electrochemical parameters relevant to microbial activity throughout the growth cycle. For circuit model development, the elements are sized using a complex non-linear squares (CNLS) method to give the best fit of the equivalent circuit model to the experimental data. Typical electric circuits used to model impedance data are resistor (zero frequency impedance, R), capacitor (C), constant phase element (CPE), and Warburg impedance (W). The relationship between the impedance and equivalent elements (Sagüés et al. 1996) are shown below.

$$ {Z}_{\mathrm{R}}=\mathrm{R} $$

$$ {Z}_{\mathrm{C}}=1/\left(j\upomega \mathrm{C}\right):j\ \mathrm{is}\ \mathrm{an}\ \mathrm{imaginary}\ \mathrm{number}\ \left(\surd -1\right) $$

$$ {Z}_{\mathrm{CPE}}=1/{\left(j\upomega \right)}^pT:p=\mathrm{phase}\ \mathrm{angle},T=\mathrm{capacitance} $$

$$ {Z}_{\mathrm{w}}=1/\mathrm{T}\surd \left(j\upomega \right) $$

**Fig. 5:**
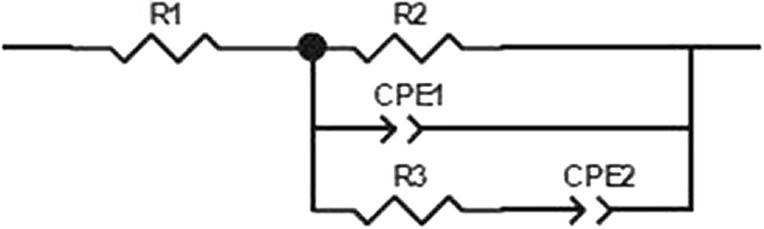
Defining microbial activity with equivalent circuits. This equivalent circuit was used to fit microbiological data where R1 represents electrolyte resistance in the medium at high frequency, CPE1 and R2 represent a constant phase element and charge transfer resistance, respectively at lower to medium frequencies, with CPE2 and R3 representing a constant phase element and a modification of the charge transfer resistance respectively at low to high frequencies

This approach was used to interpret impedance data during growth of the spore former, *Clostridium phytofermentans*, in batch assays (Martin et al. 2018) using the equivalent circuit below (Fig. 5). The equivalent circuit was used to demonstrate the link between charge transfer resistance (R3) and decreased growth rate as the carbon and energy source diminished. A strong response from R2 during the first several hours of incubation of this sporulated culture, prior to carbon source utilization was linked to the strong ionic flux during spore germination (Martin et al. 2018).

Impedance techniques for monitoring biological activity in the subsurface, including bioremediation, are also gaining interest due to the real-time data acquisition and non-invasive approach that offer significant advantages over conventional techniques. Liu et al. (2011) used a pyrite-carbon paste electrode to demonstrate the contribution of *Acidithiobacillus ferrooxidans* on accelerated pyrite oxidation using electrochemical impedance spectrometry in conjunction with X-ray photoelectron spectroscopy.

An impedance technique used in geophysics to study porous media, especially in the field, is spectral induced polarization (SIP). SIP involves injection of a current at variable frequencies into the subsurface and measuring the resulting voltage potentials received at electrodes above and below the surface (Williams et al. 2009). SIP measures complex dielectric permittivity (or complex electrical conductivity) across a spectrum of frequencies (Zhang et al. 2013). A significant value of this technique is that it can be used to link microbial processes to biogeochemical alterations in the subsurface (Mellage et al. 2018). Zhang et al. (2013) demonstrated that sulfate-reducing bacteria, a common microbe in the subsurface and capable of significant geochemical alterations, exhibit a higher dielectric response as a function of bacterial density, below frequencies of 10 kHz. Microbial activity in saturated sediments contaminated with BTEX was also reported as temporal increases in real (*σ*′) and imaginary (*σ*″) conductivity measured at 1 Hz and correlated with significant increases in bacterial density and decreases in BTEX, NO_3_ and SO_4_ (Abdel Aal et al. 2004). Microbial growth, attachment, and biofilm formation were linked most closely to complex conductivity measured at 2 Hz, especially *σ*″, and was suggested to be a proxy indicator of subsurface microbial activity (Davis et al. 2006). The use of multifrequency measurements was demonstrated through biogenic mineral transformation from bioreduction of iron and sulfate (Williams et al. 2009). In this study, microbial-induced sulfate and iron reduction were successfully tracked at 0.125 and 1 Hz over several months, following acetate injection into the subsurface. As this field advances, SIP techniques offer potential in bioremediation, enhanced oil recovery, and a possibility to measure subsurface life on other planets (Davis et al. 2006).

### Summary

The combined efforts of microbiologists and electrochemists in recent years are providing spectacular opportunities in real-time in situ analyses of bioprocesses throughout biotechnology. Overall, electrochemical techniques are providing novel biotechnologies for contaminant monitoring, waste to electricity production, and CO_2_ conversion to fuels and chemicals. Electrogenic microorganisms in particular are providing abundant opportunities for biotechnological applications that incorporate chronoamperometric and voltammetric techniques. These versatile techniques are also providing numerous analytical approaches for monitoring microbial activities in biotechnical applications. Applications including microbial fuel cells and bioelectrosynthesis are also novel bioprocessing techniques that are gaining international interest, especially for electricity generation, fuel and chemical production, and self-sustaining waste treatment. These techniques are also providing bioprocess analyses with new paradigms that will allow for real-time, in situ monitoring, which could be exploited for more efficient, automated process control. Impedance spectroscopy allows microbiologists to view microbes as electrical circuits and hence begin to redefine physiological activity in terms of electron flow through equivalent circuits and hence model microbial growth as an electrochemical phenomenon.
